# Comparison of soil eDNA to camera traps for assessing mammal and bird community composition and site use

**DOI:** 10.1002/ece3.70022

**Published:** 2024-07-14

**Authors:** Sasha J. Tetzlaff, Aron D. Katz, Patrick J. Wolff, Matthew E. Kleitch

**Affiliations:** ^1^ Engineer Research and Development Center Champaign Illinois USA; ^2^ Department of Entomology University of Illinois at Urbana‐Champaign Urbana Illinois USA; ^3^ Camp Grayling Joint Maneuver Training Center Grayling Michigan USA

**Keywords:** biological communities, environmental DNA, game animal, occupancy model, site use, wildlife survey

## Abstract

Species detections often vary depending on the survey methods employed. Some species may go undetected when using only one approach in community‐level inventory and monitoring programs, which has management and conservation implications. We conducted a comparative study of terrestrial mammal and bird detections in the spring and summer of 2021 by placing camera traps at 30 locations across a large military installation in northern Michigan, USA and testing replicate soil samples from these sites for environmental DNA (eDNA) using an established vertebrate metabarcoding assay. We detected a total of 48 taxa from both survey methods: 26 mammalian taxa (excluding humans, 24 to species and two to genus) and 22 avian taxa (21 to species and one to genus). We detected a relatively even distribution of mammalian taxa on cameras (17) and via eDNA analysis (15), with seven taxa detected from both methods. Most medium‐to‐large carnivores were detected only on cameras, whereas semi‐fossorial small mammals were detected only via eDNA analysis. We detected higher bird diversity with camera traps (18 taxa) compared to eDNA analysis (eight taxa; four taxa were detected with both methods), but cameras alone were most effective at detecting smaller birds that frequently occupy arboreal environments. We also used Bayesian spatial occupancy models for two widely distributed game species (white‐tailed deer, *Odocoileus virginianus*, and ruffed grouse, *Bonasa umbellus*) that were moderately detected with both survey methods and found species‐specific site use (occupancy) estimates were similar between cameras and eDNA analysis. Concordant with similar studies, our findings suggest that a combination of camera trap and eDNA surveys could be most useful for assessing the composition of terrestrial mammal communities. Camera traps may be most efficient for assessing bird diversity but can be complemented with eDNA analysis, particularly for species that spend considerable time on the ground.

## Graphical abstract

We conducted a comparative study of terrestrial mammal and bird detections by placing camera traps at 30 locations across a large military installation in northern Michigan, USA and testing replicate soil samples from these sites for environmental DNA (eDNA) using an established vertebrate metabarcoding assay. We detected relatively even numbers of mammalian taxa on cameras and via eDNA analysis, but many medium‐to‐large carnivores were detected only on cameras, whereas semi‐fossorial small mammals were detected only via eDNA analysis. Cameras alone were most effective at detecting smaller birds that frequently occupy arboreal environments, but eDNA analysis was a useful complementary tool for detecting larger birds that spend considerable time on the ground.

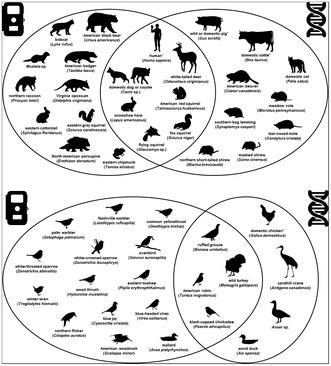

## 1 INTRODUCTION

Implementing surveys that facilitate rapid, accurate, and cost‐effective characterization of wildlife communities can be critical for management and conservation efforts (Perkins et al., 2016). However, species detections can vary depending on the survey method employed, and some species may go undetected when using only one approach in community‐level inventory and monitoring programs. Because this can influence inferences regarding species presence/absence, implementing multiple survey approaches can often lead to a more comprehensive understanding of overall species diversity across the surveyed area (Barea‐Azcón et al., 2007; Garden et al., 2007). As such, comparative assessments of survey techniques are increasingly needed, particularly those that leverage contemporary methods.

Numerous technologies exist that do not require surveyors to directly observe species to document their presence. For example, camera trap surveys have become an established method for such a purpose (Burton et al., 2015). Camera traps (i.e., game or trail cameras) provide practitioners tremendous flexibility for surveying wildlife communities with minimal disturbance. However, as with any survey method, camera traps imperfectly detect species due to numerous factors such as habitat, weather, and the angle and speed at which animals move when crossing the camera's field of view (Findlay et al., 2020; McIntyre et al., 2020). Species may also go undetected due to camera failure or theft. Additionally, observer error can occur when reviewing recorded images or videos and result in false positives via species misidentifications (Findlay et al., 2020). Further, camera traps are often inadequate for observing characters required for species‐level identifications and, like other survey methods, may present substantial bias for detecting more conspicuous groups of organisms (Johnson et al., 2023).

A more recent approach to non‐invasively survey wildlife communities is environmental DNA (eDNA) metabarcoding. This technique involves sampling environmental media such as water, soil, and air for genetic material shed by organisms that can be amplified using generalized polymerase chain reaction (PCR) primers and sequenced using next‐generation sequencing technology. Generated sequence reads are assigned to a taxonomic rank, typically within the range of order to species (Ruppert et al., 2019). eDNA metabarcoding (hereafter “metabarcoding”) has revolutionized the ability to detect numerous taxa from the same environmental samples (Deiner et al., 2017). Metabarcoding has parallels with camera trap surveys that facilitate their comparable utility for surveying wildlife communities, such as eliminating the need to directly observe taxa during surveys. eDNA analysis is not immune to issues with taxonomic detection probabilities that can occur during various stages, such as sample collection and processing (Dorazio & Erickson, 2017). Nevertheless, distinct advantages of metabarcoding are that taxonomic expertise is not required to identify taxa, and organisms need not be physically present at the time of sample collection to require detection.

Comparative studies conducted in numerous environments (e.g., lotic systems, desert springs, and artificial ponds) across multiple continents suggest that a combination of camera trap and metabarcoding surveys maximizes the detection of vertebrate taxonomic diversity. However, many studies have focused on mammals and sampled water to test for eDNA (Harper et al., 2019; Lyet et al., 2021; Mas‐Carrió et al., 2022; Mena et al., 2021; Sales et al., 2020).

Recent studies have successfully demonstrated soil eDNA analysis as an effective survey method for detecting terrestrial vertebrates in systems where water may be absent in sufficient volumes for sampling eDNA (e.g., Katz et al., 2021). However, few studies have compared soil eDNA metabarcoding with camera trapping to characterize vertebrate communities (Holm et al., 2023; Leempoel et al., 2020; Mejia et al., 2021; Ryan et al., 2022). Additionally, in some of these studies, collection of eDNA samples was spatially or temporally disjunct from camera trap surveys (e.g., Leempoel et al., 2020; Mena et al., 2021), which could influence variation in taxonomic detections based on sampling method. Furthermore, to our knowledge, no such studies have been conducted in the Upper Midwest of the United States. To address these gaps, we surveyed vertebrate communities in northern Michigan, USA by sampling for eDNA in soil and compared taxonomic detections to those from camera traps placed at the same sites and during the same season soil samples were collected. Our main objective was to understand whether diversity of detected mammals and birds generally differed between these methods. Using eDNA and camera detections for two widely distributed North American game species, we also aimed to determine whether estimates of site use for each species were dependent on sampling method.

## 2 STUDY AREA

The state of Michigan in the United States has a temperate continental climate with no dry season (Belda et al., 2014). Located in the northern Lower Peninsula of Michigan, Camp Grayling Joint Maneuver Training Center (59,488 ha) is the largest National Guard training facility in the United States. Local topography was shaped by the most recent glaciation and water bodies found on the landscape included glacial lakes, creeks, and rivers. Elevation on the installation ranges from 175 to 469 m (CGMTC, 2020). Habitat composed of coniferous and hardwood forests largely dominated the study area and contained plant species such as spruce (*Picea* spp.), cedar (*Thuja* spp.), pine (*Pinus* spp.), maple (*Acer* spp.), oak (*Quercus* spp.), and aspen (*Populus* spp.) stands; barrens dominated by lichen and blueberry (*Vaccinium* spp.); and scrub‐shrub wetlands comprised graminoids (e.g., *Carex* spp., *Eleocharis* spp.), forbs and herbs (e.g., *Matteuccia struthiopteris*), and woody species such as willow (*Salix* spp.) and speckled alder (*Alnus incana*).

## 3 METHODS

**FIGURE 1:**
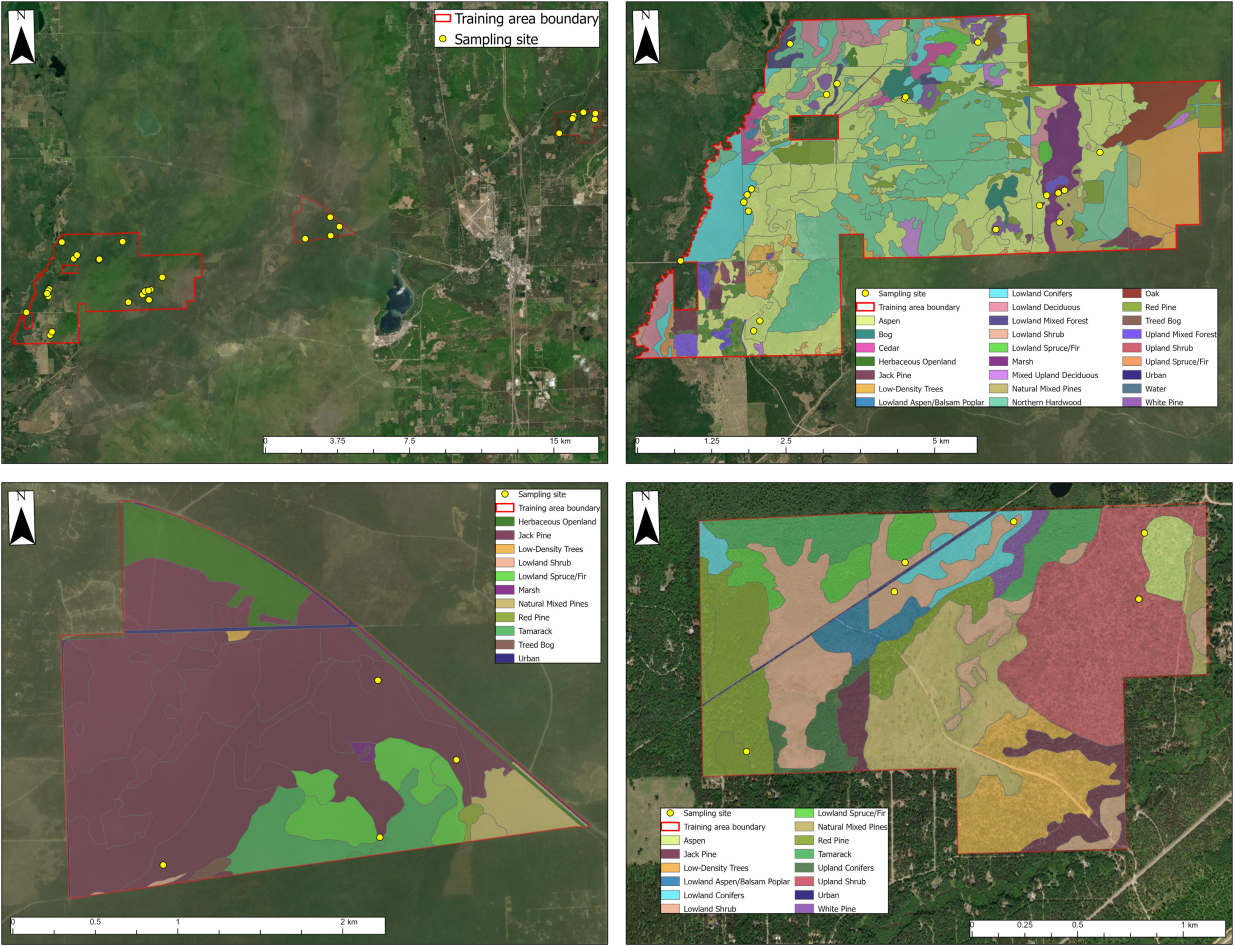
Training areas and habitat types where camera trap and environmental DNA surveys were conducted from April to September 2021 at Camp Grayling, Michigan, USA.

We sampled four, six, and 20 sites during April–September 2021 within the boundaries of three training areas on Camp Grayling that were 434, 310, and 3275 ha, respectively. These 30 sites were randomly selected using ArcGIS Pro, had habitats representative of available diversity across the installation (Figure 1), and were regularly accessible (i.e., not prone to frequent closure due to military training activities). Many sites were forested and dominated by conifers such as Jack pine (*Pinus banksiana*), red pine (*Pinus resinosa*), white cedar (*Thuja occidentalis*), tamarack (*Larix laricina*), and spruce, and hardwoods such as maple, oak, and aspen. Other sites were located on the edges of marshes and in shrublands dominated by alder (*Alnus* spp.). At each site, we placed a 3 m long × 0.5 m tall fence constructed from fabric silt (erosion) fencing. We buried fences ~10 cm into the ground and secured them in place with wooden stakes.

### 3.1 Camera trap surveys

**FIGURE 2:**
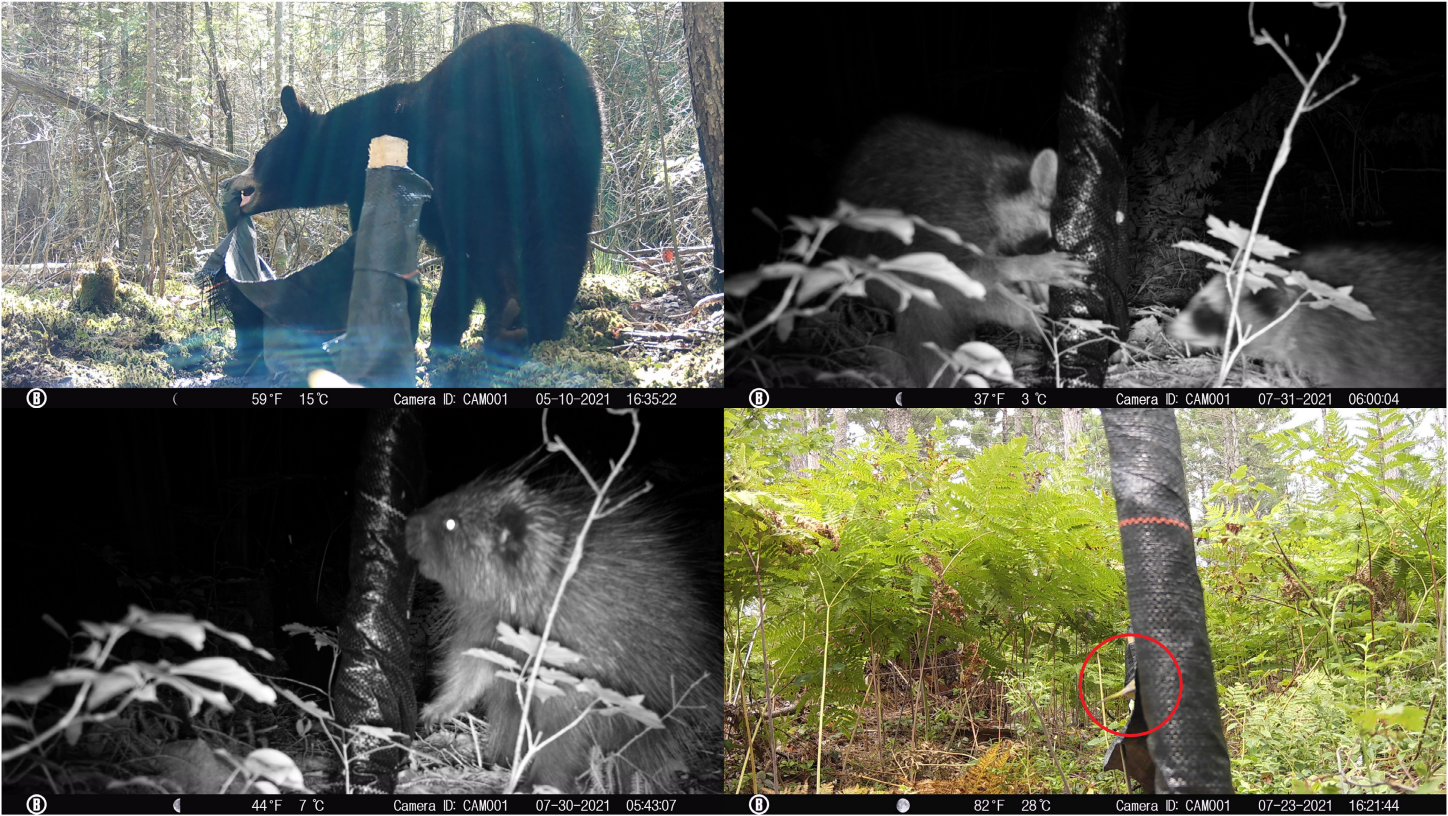
Clockwise from top‐left: American black bear (*Ursus americanus*), northern raccoon (*Procyon lotor*), unidentified passerine (circled), and North American porcupine (*Erethizon dorsatum*) interacting with fences at Camp Grayling, Michigan, USA.

We secured a Bushnell Trophy Cam (Bushnell Outdoor Products, Overland Park, Kansas, USA) to a wooden stake that was aimed at one end of each fence and programmed to take a photograph when its motion sensor was triggered. The fences were intended to aid in capturing images of herpetofauna (reptiles and amphibians), but we did not observe any such taxa when reviewing camera images. However, we suspect the fences facilitated capturing images of mammals and birds by serving as novel attractants in the environment. For instance, mammals frequently interacted with fences, and birds used them as perches (Figure 2). Cameras were generally available to detect wildlife from 29 April to 15 September 2021, although three cameras placed in riparian areas failed due to flooding. Additionally, one camera was tampered with by an American black bear (*Ursus americanus*); data from this camera were used to determine overall mammal and bird diversity but not for occupancy analyses (see below) because the date formatting was altered (many images had a date of 1900 when reviewing camera media). All photos were reviewed and annotated using the image analysis software Timelapse 2 (Greenberg, 2021) to assign species identities, when possible. A single observer initially tagged all camera trap images, and two of us (SJT and PJW) independently verified species' identities for a subset of tagged photos (*n* = 15,442) where the initial observer was uncertain; we solicited expert opinion in limited cases where we were uncertain of a species' identity.

### 3.2 Environmental DNA surveys

We conducted three rounds of soil sampling to test for vertebrate eDNA that occurred during 30 April–3 May, 12–14 July, and 13–15 September. Due to site access issues, we were unable to sample three sites during the second round and one site in the third. When sampling each site, we collected ~4 L of soil from both sides of each fence into a sterile Ziploc® bag. We also collected control soil samples in a similar fashion at a local hotel that was approximately 8, 10, and 18 km straight‐line distance from each of the training areas we sampled in on Camp Grayling, but there were no fences at these sites. We then homogenized each bag and transferred 50 mL of soil into a sterile Falcon® tube and froze samples until further processing.

We subsampled 0.25 g of soil in triplicate for each sample. eDNA was then isolated from each subsample, including field negative controls, following the muDNA DNA extraction protocol for soil samples (Sellers et al., 2018). A two‐step PCR protocol was used to construct metabarcoding libraries following the Illumina 16S metagenomic sequencing library preparation guidelines, modified for vertebrate 12S rRNA using vertebrate‐specific primers (12S‐V5) developed by Rias et al. (2011). Vertebrate, locus‐specific primers, modified with Illumina overhang adapter sequences, were used for the first PCR (PCR1) (Table 1). Each 25 μL PCR1 reaction was performed in triplicate for all samples, negative controls (sterile, molecular grade water), and positive controls (European glass lizard [*Pseudopus apodus*] DNA template [0.05 ng/μL]). Each PCR1 reaction used 25‐μL volumes, consisting of 3 μL of DNA template, 12.5 μL of KAPA HiFi HotStart ReadyMix (Roche Sequencing), 0.4 μL of bovine serum albumin (BSA), 6.1 μL of sterile, molecular grade water, and 1.5 μL of each primer (10 μM). Thermocycling conditions for PCR1 included a 98°C incubation step for 5 min, followed by 35 cycles of 98°C for 10 s, 58°C for 30 s, and 72°C for 30 s. Triplicate reactions for each sample were pooled and amplification was verified via gel electrophoresis. PCR1 products (55 μL) were size‐selected and cleaned with Ampure XP beads (Beckman Coulter) and confirmed via gel electrophoresis.

**TABLE 1:** Forward and reverse PCR1 primers and PCR2 indexes, including name, length, and original references, for the 12S vertebrate eDNA metabarcoding assay used in this study.

Name	Sequence (5′–3′)	References
12S‐V5 Forward	**TCGTCGGCAGCGTCAGATGTGTATAAGAGACAG**ACTGGGATTAGATACCCC	Riaz et al. (2011)
12S‐V5 Reverse	**GTCTCGTGGGCTCGGAGATGTGTATAAGAGACAG**TAGAACAGGCTCCTCTAG	Riaz et al. (2011)
Index 1	CAAGCAGAAGACGGCATACGAGAT[10bp_Index]GTCTCGTGGGCTCGG	IDT‐Illumina UD i7 Indexes
Index 2	AATGATACGGCGACCACCGAGATCTACAC[10bp_Index]TCGTCGGCAGCGTC	IDT‐Illumina UD i5 Indexes

*Note*: PCR1 Illumina adaptor sequences are highlighted in bold.

In the second PCR (PCR2), 10 bp unique dual indexes were added to the amplified products using IDT for Illumina UD Indexes (Table 1). PCR2 was performed in duplicate for each sample, including negative and positive controls. Each PCR2 reaction used 25‐μL volumes, consisting of 2 μL of PCR1 template, 12.5 μL of KAPA HiFi HotStart ReadyMix, 5.5 μL of sterile, molecular grade water, and 5 μL of IDT for Illumina Unique Dual indexes (10 μM). Thermocycling conditions for PCR2 included a 95°C incubation step for 3 min, followed by 10 cycles of 98°C for 30 s, 55°C for 30 s, and 72°C for 30 s. Duplicate reactions for each sample were pooled and amplification was verified via gel electrophoresis. PCR2 products (35 μL) were size‐selected and cleaned with Ampure XP beads and confirmed via gel electrophoresis. PCR2 samples were normalized and pooled into sub‐libraries by plate based on DNA concentrations determined using the Qubit dsDNA HS kit (Invitrogen). Ampure XP bead cleanups were performed again on each sub‐library to concentrate DNA and ensure primer dimer removal prior to sequencing. Sub‐libraries were then normalized, pooled, and submitted to Azenta‐Genewiz (South Plainfield, NJ) for sequencing on the HiSeq platform for 2 × 150 bp paired‐end reads, with an output of 450 million reads.

Demultiplexed sequences were trimmed to remove adaptors from both 5′ and 3′ ends of each forward and reverse read using cutadapt (Martin, 2011) in Qiime2 v 2022.2 (Bolyen et al., 2019) and amplicon sequencing variants (ASVs) were generated using the DADA2 denoise function (Callahan et al., 2016) in Qiime2. Taxonomic assignments for each ASV were applied with the Qiime2 feature classifier classify‐sklearn function (Pedregosa et al., 2011) using a naive Bayes classifier trained on all unique 12S sequences for vertebrate taxa reported in Michigan available on NCBI GenBank (*n* = 781 sequences representing 522 species). To control for contamination, the maximum number of reads for each ASV found in field, extraction, and PCR controls were removed from all the other samples. All taxonomic classifications were confirmed via NCBI BLASTn search against the full GenBank nucleotide database. ASVs were considered misclassified if BLAST returned different identifications to species that occur in Michigan with greater than or equal to 98% pairwise identity. If more than one species was matched following this criterion, then the ASV was left identified to genus or family. ASVs were then combined by classification to generate a list of taxa and associated read counts for each sample. As we describe in detail below, all taxa were identified at least to genus, but most were identified to species.

### 3.3 Data analyses

We conducted statistical analyses using R version 4.2.2 (R Core Team, 2022). We used a generalized linear model (GLM) assuming a Poisson distribution to determine if the total number of taxa detected per site differed between survey methods. We repeated this analysis using only the number of mammalian and avian taxa detected per site as the response variable to explore whether results from our initial GLM may have been driven by either group. We ensured data were not overdispersed in any of the three models using the *dispersiontest* function in the *AER* package (Kleiber & Zeileis, 2008) and found no violations (all *p* > .14). We generated predicted means (estimated marginal means) for each sampling method using the *emmeans* package (Lenth, 2023).

Ruffed grouse (*Bonasa umbellus*) and white‐tailed deer (*Odocoileus virginianus*) are two game species that were both moderately detected during this study with cameras and metabarcoding. To determine whether estimates of site use were comparable between sampling methods, we used detection/non‐detection data for these species from camera traps and replicate soil samples to simultaneously estimate occupancy (*p*_si_: the probability that the species is present at site *i*) and detection probability (*p*: the probability that the species is detected at time *i* at site *j*, given it was present at site *j*; MacKenzie et al., 2002). We used “NAs” in the detection/non‐detection datasets for days when cameras were not available to detect animals (e.g., due to malfunction from site flooding as described above). An assumption of single‐season occupancy models is that sites are “closed”, meaning the occupancy status for a species at a given site should not change between surveys. Because this assumption was violated for both camera and eDNA surveys, we interpreted estimates of *p*_si_ as the probability of site use rather than occupancy (sensu Kovalenko et al., 2024).

We used the *spOccupancy* package (Doser et al., 2022) to fit spatially explicit occupancy models in a Bayesian framework using Pólya‐Gamma data augmentation and nearest‐neighbor Gaussian processes to account for potential spatial dependence (autocorrelation) among observations in detection/non‐detection data (Johnson et al., 2013). When constructing models, we set the hypermeans to 0 and the hypervariances to 2.72 (which correspond to relatively flat priors on the probability scale) and specified two chains with 5000 samples per chain, a burn‐in of 3000, and a thinning rate of two for the Markov Chain Monte Carlo (MCMC) algorithm. We ensured adequate mixing of the MCMC chains occurred by inspecting Rhat values (which were <1.1 for occupancy and detection estimates) and visually assessing trace plots. We ran intercept‐only models (i.e., models with no covariates) to estimate naïve occupancy (site use) and detection probabilities with 89% credible intervals (McElreath, 2018). We conducted posterior predictive checks of models by computing Bayesian *p*‐values using the Freeman–Tukey statistic in *spOccupancy*, which ranged from 0.19 to 0.48, suggesting no lack of fit.

## 4 RESULTS

**FIGURE 3:**
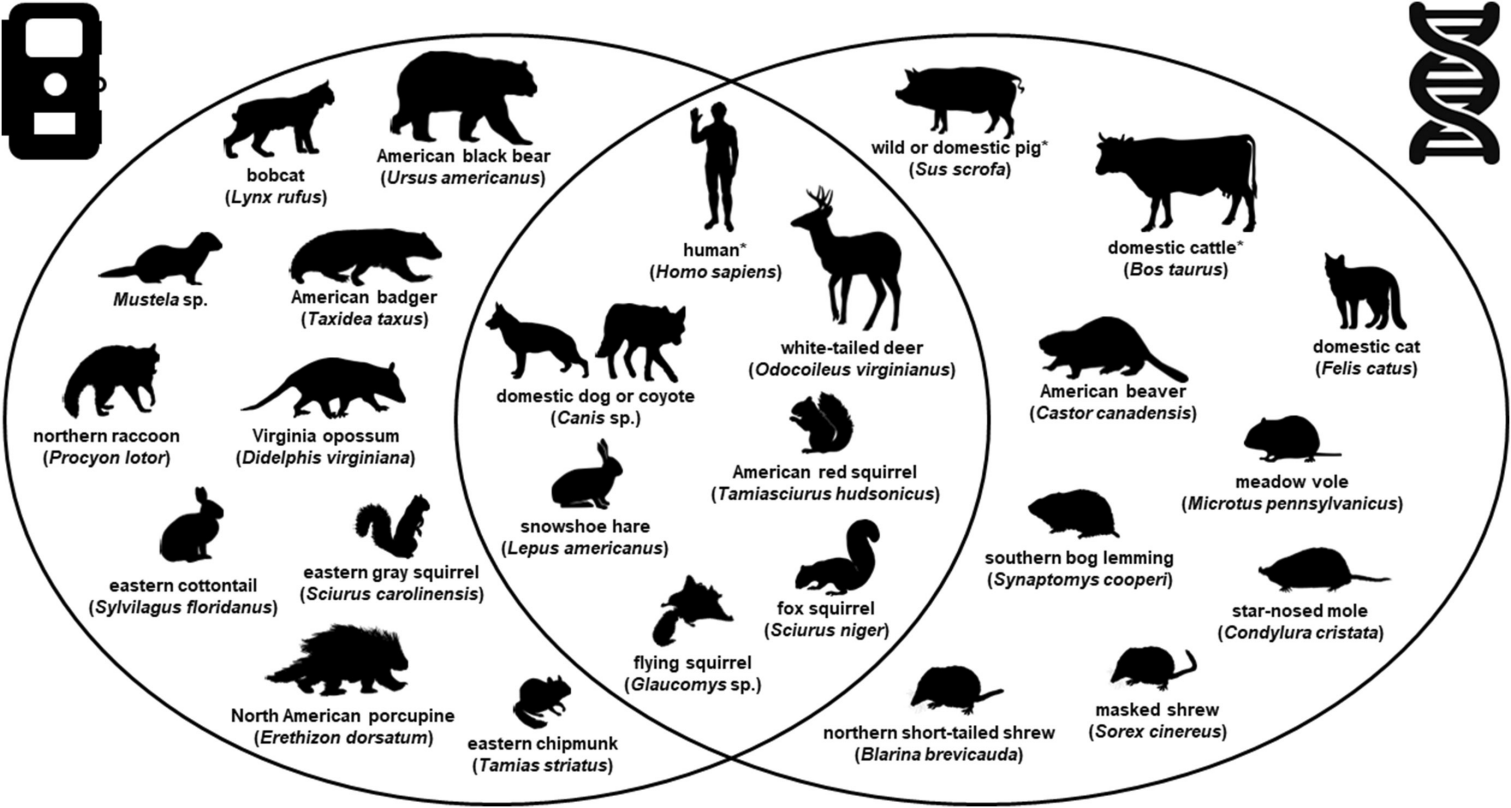
Venn diagram of mammalian taxa detected with camera traps (left), environmental DNA from soil samples (right), or both survey methods (middle) on Camp Grayling, Michigan, USA, from April to September 2021. Note that coyote (*Canis latrans*) and domestic dog (*Canis lupus familiaris*) could not be distinguished via eDNA analysis because their reference sequences are identical for the targeted 12S gene region, but because we detected each species on cameras, we considered them separately in our total mammalian taxonomic detections. Southern flying squirrel (*Glaucomys volans*) was identified via eDNA analysis, but we could not distinguish whether flying squirrels detected on cameras were northern flying squirrel (*Glaucomys sabrinus*) or southern flying squirrel, so we conservatively considered *Glaucomys* sp. as one taxon in our total mammalian taxonomic detections. *Indicates possible taxonomic detection based on contamination. Credits for each illustration are provided in Data S1.

We detected a total of 48 taxa from both survey methods. Excluding humans, we detected 26 mammalian taxa; we identified 24 to species and two to genus (see below). We detected 10 mammalian taxa only on cameras (17 overall), nine with only eDNA analysis (15 overall), and seven with both methods, excluding human detections (Figure 3). We detected coyote (*Canis latrans*) and domestic dog (*Canis lupus familiaris*) on cameras and thus considered these species separately in our total mammalian taxonomic detections, but these species could not be distinguished via eDNA analysis because their reference sequences are identical for the targeted 12S gene region. Southern flying squirrel (*Glaucomys volans*) was identified via eDNA analysis, and although we detected flying squirrels on cameras, we could not distinguish northern flying squirrel (*Glaucomys sabrinus*) from southern flying squirrel. We thus conservatively considered *Glaucomys* sp. as one taxon in our total mammalian taxonomic detections. Most medium‐to‐large carnivores such as American black bear, bobcat (*Lynx rufus*), and American badger (*Taxidea taxus*) were detected only on cameras. Conversely, semi‐fossorial small mammals such as meadow vole (*Microtus pennsylvanicus*), southern bog lemming (*Synaptomys cooperi*), star‐nosed mole (*Condylura cristata*), and shrews (*Blarina* and *Sorex*) were detected only via eDNA analysis. All mammalian taxa had been previously documented on Camp Grayling, aside from bobcat, Virginia opossum (*Didelphis virginiana*), and star‐nosed mole (*Condylura cristata*) (CGMTC, 2020). Bobcat and Virginia opossum were confirmed detected only from camera traps, and star‐nosed mole was detected only via eDNA analysis.

**FIGURE 4:**
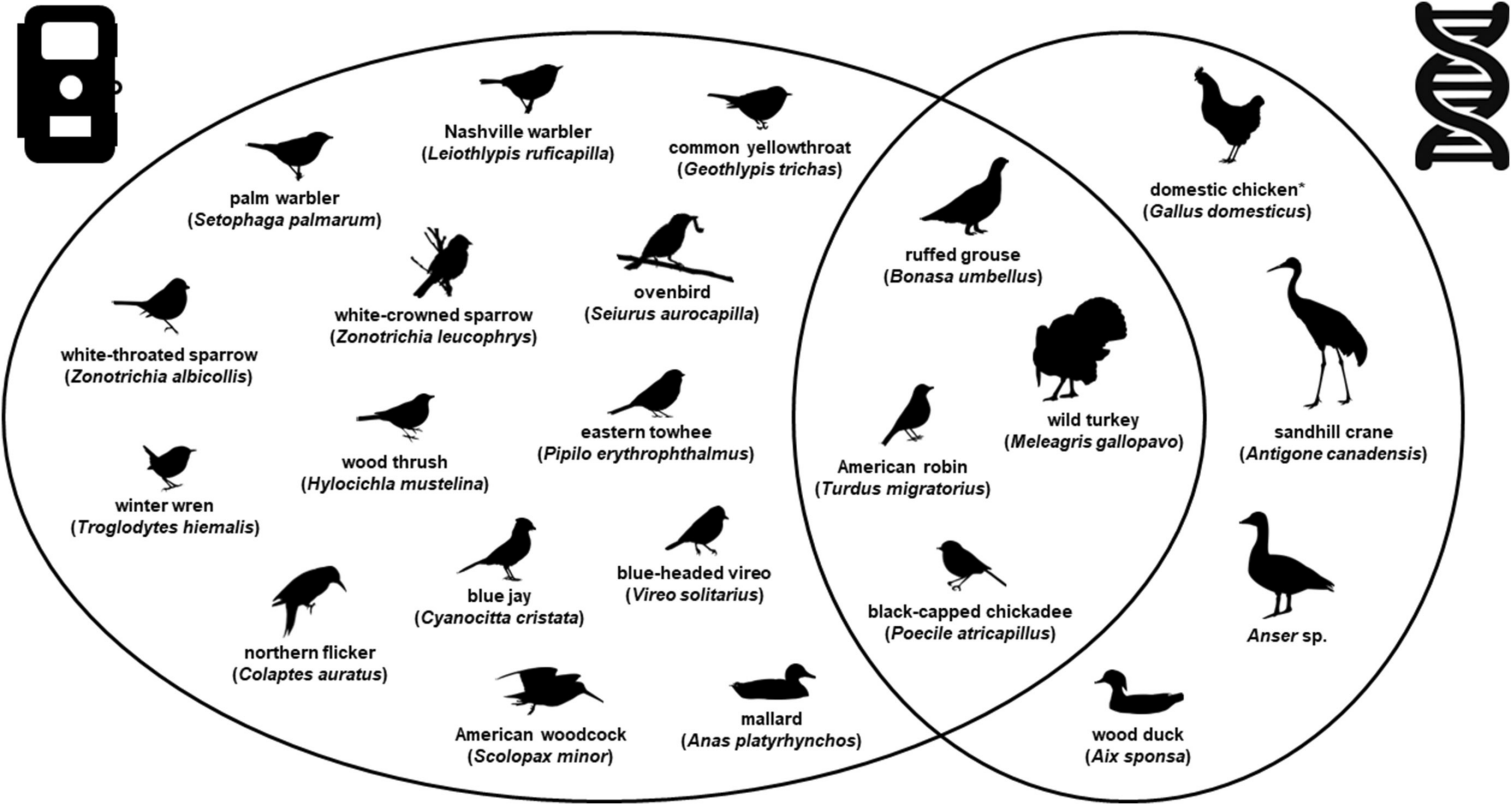
Venn diagram of avian taxa detected with camera traps (left), environmental DNA from soil samples (right), or both survey methods (middle) on Camp Grayling, Michigan, USA, from April to September 2021. *Indicates possible taxonomic detection based on contamination. Credits for each illustration are provided in Data S1.

We detected a total of 22 avian taxa. We detected 14 avian taxa only on cameras (18 overall), four with only eDNA analysis (eight overall), and four with both methods (Figure 4). Aside from one taxon that was identified to genus (*Anser* [geese], detected with eDNA analysis), all other taxa were identified to species with both methods. Cameras alone were most effective at detecting smaller passerines that frequently occupy arboreal environments. Conversely, eDNA analysis alone was effective at detecting larger birds that spend considerable time terrestrially or in aquatic habitats, such as sandhill crane (*Antigone canadensis*), wood duck (*Aix sponsa*), and *Anser* geese. All avian taxa had been previously documented on Camp Grayling, aside from blue‐headed vireo (*Vireo solitarius*) and palm warbler (*Setophaga palmarum*), which were confirmed detected only from camera traps during this study (CGMTC, 2020).

**FIGURE 5:**
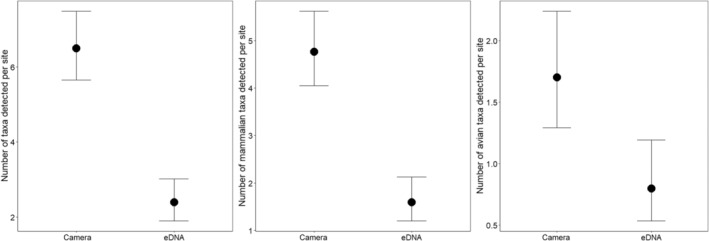
Mean with 95% confidence interval number of taxa (overall, mammalian, and avian) detected per site at Camp Grayling, Michigan, USA based on sampling method (camera trap or environmental DNA analysis).

We found evidence to suggest the mean number of taxa detected per site was lower using eDNA analysis than cameras (β = −0.996, 95% confidence interval [CI]: −1.272 to −0.731) (Figure 5). On average, cameras were predicted to detect approximately three times the number of taxa per site than with eDNA analysis. We found similar evidence when restricting the data to only mammal (−1.092, 95% CI: −1.428 to −0.773) or bird (−0.754, 95% CI: −1.256 to −0.280) detections. However, the difference in the mean number of mammalian taxa detected per site based on sampling method was greater than it was for birds (Figure 5).

Of the 29 sites included for occupancy modeling, white‐tailed deer were detected on cameras at 22 sites and via eDNA at 19 sites. Estimated naïve occupancy and detection probability for white‐tailed deer on camera traps was 0.77 (89% CrI: 0.61–0.88) and 0.10 (89% CrI: 0.09–0.11), respectively. Estimated naïve occupancy and detection probability for white‐tailed deer eDNA were 0.78 (89% CrI: 0.56–0.92) and 0.50 (89% CrI: 0.38–0.63), respectively.

Ruffed grouse were detected on cameras at 12 sites and via eDNA at 10 sites. Estimated naïve occupancy and detection probability for ruffed grouse on camera traps were 0.50 (89% CrI: 0.30–0.71) and 0.02 (89% CrI: 0.01–0.03), respectively. Estimated naïve occupancy and detection probability for ruffed grouse eDNA were 0.45 (89% CrI: 0.23–0.75) and 0.41 (89% CrI: 0.23–0.60), respectively.

## 5 DISCUSSION

Concordant with similar studies, our overall findings suggest that a combination of camera trap and eDNA surveys could be most useful for assessing the composition of mammal and bird communities (Harper et al., 2019; Holm et al., 2023; Lyet et al., 2021; Mas‐Carrió et al., 2022; Mejia et al., 2021). However, we detected differences in taxonomic detections that were likely based on factors such as body size and behavior (Andersen et al., 2012). For example, we detected many medium‐to‐large mammals only on cameras, whereas semi‐fossorial small mammals were detected only via eDNA analysis. Similar findings were reported by Mena et al. (2021) for terrestrial mammals in Peruvian tropical forests and Harper et al. (2019) for terrestrial and semi‐aquatic mammals at natural ponds in Europe. These findings cumulatively suggest that home range size, which tends to increase with body size (Swihart et al., 1988), can influence likelihood of detecting various mammals. Although semi‐fossorial small mammals may have generally low detection probabilities on cameras due to their small body size and cryptic nature compared to larger and more mobile mammals, we suspect that more eDNA may be detectable for these taxa when sampling sites that occur within their relatively smaller home ranges. Additionally, eDNA deposited by semi‐fossorial taxa into soil may be more protected from environmental factors that degrade DNA, such as exposure to ultraviolet radiation and comparatively higher surface soil temperatures (Katz et al., 2021; but see Guthrie et al., 2024). Conversely, larger mammals are more conspicuous and thus likely have higher detection probabilities on cameras but may not deposit suitable quantities of eDNA for detection if they spend limited time at a sampling site while traversing larger home ranges.

Differences in behavior and body size also appeared to influence avian detections based on sampling method. For instance, most bird species we detected only on cameras were small passerines that frequently occupy arboreal environments and are highly mobile. Although we detected some of these species on the ground when reviewing camera images, they were likely not detected via eDNA analysis for reasons similar to the aforementioned presumed detection issues with larger mammals. These results suggest that camera traps may be most efficient for assessing bird diversity but can be complemented with eDNA analysis, particularly for larger species that spend considerable time on the ground and thus likely deposit more eDNA that is available for detection.

Our surveys led to the detection of several species of conservation concern. Bird species we detected on cameras such as wood thrush (*Hylocichla mustelina*), northern flicker (*Colaptes auratus*), ruffed grouse, Nashville warbler (*Leiothlypis ruficapilla*), common yellowthroat (*Geothlypis trichas*), and white‐throated sparrow (*Zonotrichia albicollis*) are listed as priorities in the Partners in Flight, Bird Conservation Plan for The Boreal Hardwood Transition (Matteson et al., 2009). Additionally, wood thrush is a Department of Defense (DoD), Tier 2 Mission‐Sensitive Species (DoD MSS, 2021). Although we detected southern flying squirrel eDNA in soil, we could not distinguish northern from southern flying squirrels on camera images. Northern flying squirrels are a species of special concern in Michigan, but southern flying squirrels are not (MNFI, 2024). Both species have been reported on Camp Grayling (CGMTC, 2020), so understanding their distribution across the installation is important for management. In such cases where taxonomic detections are limited to genus or higher, single‐species quantitative polymerase chain reaction (qPCR) assays, or a different metabarcoding approach that targets a more variable genetic marker to distinguish the species of interest may be needed to clarify questionable species identifications (Harper et al., 2018).

Although comparative survey approaches such as those we used here are useful for determining taxonomic diversity, detection/non‐detection data from such surveys has been increasingly used to estimate species distributions with occupancy models (DeGregorio et al., 2023; Katz et al., 2023). Results from our occupancy analyses for white‐tailed deer and ruffed grouse suggest that using either camera or eDNA surveys could be independently sufficient for modeling their distributions. Differences in detection probabilities between survey method for each species can be attributed to sampling scheme, given only three eDNA samples were taken compared to essentially daily detection probabilities for camera traps. A potential benefit of using eDNA analysis over cameras for such purposes is that only three samples per site were needed to generate estimates of site use, whereas cameras were active across the entire survey period. Future research investigating how much sampling effort would be needed and estimated cost for each survey method to achieve similar estimates of site use could be useful.

## 6 RESEARCH IMPLICATIONS

Military personnel rely on realistic training environments to achieve mission priorities. Consequently, military installations tend to have large swaths of land that can be quality wildlife habitat and support high biodiversity (Aycrigg et al., 2015; Stein et al., 2008; Zentelis & Lindenmayer, 2015). Therefore, many United States Department of Defense installations are required by national law to have an Integrated Natural Resource Management Plan (INRMP) that outlines how significant natural resources are managed. An important component of INRMPs is a comprehensive list of species documented on the installation (notably vertebrates, invertebrates, and plants), which is enhanced through baseline surveys like we conducted here. However, our approach is not limited to DoD lands and has broad utility for those interested in how taxonomic detections from camera traps and soil eDNA analysis compare.

Deciding on what survey method(s) to employ depends on a variety of factors, including resource availability, experience, expertise, and the objectives of the inventory or monitoring program. Each survey method we used has pros and cons. Although camera traps are relatively inexpensive and easy to set up and maintain, considerable time can be invested in reviewing images or videos to identify species. However, advances in artificial intelligence such as machine learning are helping to dramatically decrease the time, effort, and cost involved with identifying species from camera media (Tabak et al., 2019). eDNA surveys generally entail rapid sample collection but rely on expertise with laboratory practices for sample processing.

If the main goal is to maximize the overall number of detected taxa, our findings suggest that a combination of camera trap and eDNA surveys could be most beneficial. However, when examining taxonomic detections on a per site level, cameras were superior to eDNA analysis for overall detections and mammals and birds independently. If resources are limited and only one method can be employed, camera traps may currently provide the most utility but come at the expense of not detecting some taxa (e.g., semi‐fossorial small mammals detected with only eDNA analysis) depending on camera trap placement (e.g., we placed camera traps to broadly survey community‐level diversity, not to target particular species or guilds). Replicating these kinds of comparative studies across numerous environments will be most informative for enhancing species management and conservation.

## AUTHOR CONTRIBUTIONS

**Sasha J. Tetzlaff:** Conceptualization (lead); data curation (equal); formal analysis (lead); funding acquisition (lead); investigation (equal); methodology (equal); project administration (equal); visualization (lead); writing – original draft (lead); writing – review and editing (lead). **Aron D. Katz:** Conceptualization (supporting); data curation (equal); funding acquisition (equal); investigation (equal); methodology (equal); writing – review and editing (equal). **Patrick J. Wolff:** Conceptualization (equal); data curation (equal); funding acquisition (equal); investigation (equal); methodology (equal); project administration (equal); supervision (equal); writing – review and editing (equal). **Matthew E. Kleitch:** Conceptualization (equal); data curation (equal); funding acquisition (equal); investigation (equal); methodology (equal); project administration (equal); supervision (equal); writing – review and editing (equal).

## CONFLICT OF INTEREST STATEMENT

The authors declare no conflict of interest.

## Supporting information


Data S1:

## Data Availability

The data and an R script used to conduct analyses in this study are provided in the Data S1.

## References

[ece370022-bib-0001] Andersen, K. , Bird, K. L. , Rasmussen, M. , Haile, J. , Breuning‐Madsen, H. , Kjaer, K. H. , Orlando, L. , Gilbert, M. T. P. , & Willerslev, E. (2012). Meta‐barcoding of ‘dirt’ DNA from soil reflects vertebrate biodiversity. Molecular Ecology, 21, 1966–1979. 21917035 10.1111/j.1365-294X.2011.05261.x

[ece370022-bib-0002] Aycrigg, J. L. , Belote, R. T. , Dietz, M. S. , Aplet, G. H. , & Fischer, R. A. (2015). Bombing for biodiversity in the United States: Response to Zentelis & Lindenmayer. Conservation Letters, 8, 306–307.

[ece370022-bib-0003] Barea‐Azcón, J. M. , Virgós, E. , Ballesteros‐Duperón, E. , Moleón, M. , & Chirosa, M. (2007). Surveying carnivores at large spatial scales: A comparison of four broad‐applied methods. Biodiversity and Conservation, 16, 1213–1230.

[ece370022-bib-0004] Belda, M. , Holtanová, E. , Halenka, T. , & Kalvová, J. (2014). Climate classification revisited: From Köppen to Trewartha. Climate Research, 59, 1–13.

[ece370022-bib-0005] Bolyen, E. , Rideout, J. R. , Dillon, M. R. , Bokulich, N. A. , Abnet, C. C. , Al‐Ghalith, G. A. , Alexander, H. , Alm, E. J. , Arumugam, M. , Asnicar, F. , Bai, Y. , Bisanz, J. E. , Bittinger, K. , Brejnrod, A. , Brislawn, C. J. , Brown, C. T. , Callahan, B. J. , Caraballo‐Rodríguez, A. M. , Chase, J. , … Caporaso, J. G. (2019). Reproducible, interactive, scalable and extensible microbiome data science using QIIME 2. Nature Biotechnology, 37, 852–857. 10.1038/s41587-019-0209-9 PMC7015180 31341288

[ece370022-bib-0006] Burton, A. C. , Neilson, E. , Moreira, D. , Ladle, A. , Steenweg, R. , Fisher, J. T. , Bayne, E. , & Boutin, S. (2015). Wildlife camera trapping: A review and recommendations for linking surveys to ecological processes. Journal of Applied Ecology, 52, 675–685.

[ece370022-bib-0007] Callahan, B. J. , McMurdie, P. J. , Rosen, M. J. , Han, A. W. , Johnson, A. J. A. , & Holmes, S. P. (2016). DADA2: High‐resolution sample inference from Illumina amplicon data. Nature Methods, 13, 581–583. 27214047 10.1038/nmeth.3869 PMC4927377

[ece370022-bib-0008] Camp Grayling Maneuver Training Center (CGMTC) . (2020). Integrated natural resources management plan . 251 pp.

[ece370022-bib-0009] DeGregorio, B. A. , McElroy, M. R. , & Johansson, E. P. (2023). Occupancy and activity patterns of nine‐banded armadillos (*Dasypus novemcinctus*) in a suburban environment. Diversity, 15, 907.

[ece370022-bib-1002] Deiner, K. , Bik, H. M. , Mächler, E. , Seymour, M. , Lacoursiere‐Roussel, A. , Altermatt, F. , Creer, S. , Bista, I. , Lodge, D. M. , de Vere, N. , Pfrender, M. E. , & Bernatchez, L. (2017). Environmental DNA metabarcoding: Transforming how we survey animal and plant communities. Molecular Ecology, 26, 5872–5895. 28921802 10.1111/mec.14350

[ece370022-bib-0010] Department of Defense, Mission‐Sensitive Species (MSS) . (2021). https://www.denix.osd.mil/dodpif/denix‐files/sites/37/2023/12/DoD‐PIF‐MSS‐Fact‐Sheet_508_v2.pdf

[ece370022-bib-0011] Dorazio, R. , & Erickson, R. (2017). *eDNAoccupancy*: An R package for multi‐scale occupancy modeling of environmental DNA data. Molecular Ecology Resources, 19, 368–380. 10.1111/1755-0998.12735 29120090

[ece370022-bib-0012] Doser, J. W. , Finley, A. O. , Kéry, M. , & Zipkin, E. F. (2022). *spOccupancy*: An R package for single‐species, multi‐species, and integrated spatial occupancy models. Methods in Ecology and Evolution, 13, 1670–1678.

[ece370022-bib-0013] Findlay, M. A. , Briers, R. A. , & White, P. J. C. (2020). Component processes of detection probability in camera‐trap studies: Understanding the occurrence of false‐negatives. Mammal Research, 65, 167–180.

[ece370022-bib-0014] Garden, J. G. , McAlpine, C. A. , Possingham, H. P. , & Jones, D. N. (2007). Using multiple survey methods to detect terrestrial reptiles and mammals: What are the most successful and cost‐efficient combinations? Wildlife Research, 34, 218–227.

[ece370022-bib-0015] Greenberg, S. (2021). Timelapse2 (2.2.3.9) [windows] . https://saul.cpsc.ucalgary.ca/timelapse/pmwiki.php?n=Main.HomePage

[ece370022-bib-1006] Guthrie, A. M. , Cooper, C. E. , Bateman, P. W. , van der Heyde, M. , Allentoft, M. E. , & Nevill, P. (2024). A quantitative analysis of vertebrate environmental DNA degradation in soil in response to time, UV light, and temperature. Environmental DNA, 6(4), e581.

[ece370022-bib-0016] Harper, L. R. , Handley, L. L. , Hahn, C. , Boonham, N. , Rees, H. C. , Gough, K. C. , Lewis, E. , Adams, I. P. , Brotherton, P. , Phillips, S. , & Hänfling, B. (2018). Needle in a haystack? A comparison of eDNA metabarcoding and targeted qPCR for detection of the great crested newt (*Triturus cristatus*). Ecology and Evolution, 8, 6330–6341. 29988445 10.1002/ece3.4013 PMC6024127

[ece370022-bib-0017] Harper, L. R. , Handleya, L. L. , Carpenterc, A. I. , Ghazalid, M. , Di Muria, C. , Macgregore, C. J. , Logana, T. W. , Lawf, A. , Breithaupta, T. , Readg, D. S. , McDevitt, A. D. , & Hänfling, B. (2019). Environmental DNA (eDNA) metabarcoding of pond water as a tool to survey conservation and management priority mammals. Biological Conservation, 238, 108225.

[ece370022-bib-0018] Holm, A. M. R. , Knudsen, S. W. , Månsson, M. , Pedersen, D. E. , Nordfoss, P. H. , Johansson, D. K. , Gramsbergen, M. , Havmøller, R. W. , Sigsgaard, E. E. , Thomsen, P. F. , Olsen, M. T. , & Møller, P. R. (2023). Holistic monitoring of freshwater and terrestrial vertebrates by camera trapping and environmental DNA. Environmental DNA, 5, 1608–1622. 10.1002/edn3.481

[ece370022-bib-0019] Johnson, D. S. , Conn, P. B. , Hooten, M. B. , Ray, J. C. , & Pond, B. A. (2013). Spatial occupancy models for large data sets. Ecology, 94, 801–808.

[ece370022-bib-0020] Johnson, M. D. , Katz, A. D. , Davis, M. A. , Tetzlaff, S. , Edlund, D. , Tomczyk, S. , Molano‐Flores, B. , Wilder, T. , & Sperry, J. H. (2023). Environmental DNA metabarcoding from flowers reveals arthropod pollinators, plant pests, parasites, and potential predator–prey interactions while revealing more arthropod diversity than camera traps. Environmental DNA, 5, 551–569.

[ece370022-bib-0021] Katz, A. D. , Harper, L. R. , Sternhagen, E. C. , Pearce, S. E. , Melder, C. A. , Sperry, J. H. , & Davis, M. A. (2021). Environmental DNA is effective in detecting the federally threatened Louisiana Pinesnake (*Pituophis ruthveni*). Environmental DNA, 3, 409–425.

[ece370022-bib-0022] Katz, A. D. , Tetzlaff, S. J. , Johnson, M. D. , & Sperry, J. H. (2023). Molecular identification and environmental DNA detection of gill lice ectoparasites associated with brook trout population declines. Transactions of the American Fisheries Society, 152, 788–808.

[ece370022-bib-0023] Kleiber, C. , & Zeileis, A. (2008). Applied econometrics with R. Springer‐Verlag. https://CRAN.R‐project.org/package=AER

[ece370022-bib-0024] Kovalenko, V. , Doser, J. W. , Bate, L. J. , & Six, D. L. (2024). Paired acoustic recordings and point count surveys reveal Clark's nutcracker and whitebark pine associations across glacier National Park. Ecology and Evolution, 14, e10867. 38274862 10.1002/ece3.10867 PMC10808773

[ece370022-bib-0025] Leempoel, K. , Herbert, T. , & Hadly, E. (2020). A comparison of eDNA to camera trapping for assessment of terrestrial mammal diversity. Proceedings of the Royal Society B, 287, 20192353. 31937227 10.1098/rspb.2019.2353 PMC7003463

[ece370022-bib-0026] Lenth, R. (2023). Emmeans: Estimated marginal means, aka least‐squares means . R package version 1.8.7, https://CRAN.R‐project.org/package=emmeans

[ece370022-bib-0027] Lyet, A. , Pellissier, L. , Valentini, A. , Dejean, T. , Hehmeyer, A. , & Naidoo, R. (2021). eDNA sampled from stream networks correlates with camera trap detection rates of terrestrial mammals. Scientific Reports, 11, 11362. 34131168 10.1038/s41598-021-90598-5 PMC8206079

[ece370022-bib-0028] MacKenzie, D. I. , Nichols, J. D. , Lachman, G. B. , Droege, S. , Royle, J. A. , & Langtimm, C. A. (2002). Estimating site occupancy rates when detection probabilities are less than one. Ecology, 83, 2248–2255.

[ece370022-bib-0029] Martin, M. (2011). Cutadapt removes adapter sequences from high‐throughput sequencing reads. EMBnet.Journal, 17, 10–12.

[ece370022-bib-0030] Mas‐Carrió, E. , Schneider, J. , Nasanbat, B. , Ravchig, S. , Buxton, M. , Nyamukondiwa, C. , Stoffel, C. , Augugliaro, C. , Ceacero, F. , Taberlet, P. , Glaizot, O. , Christe, P. , & Fumagalli, L. (2022). Assessing environmental DNA metabarcoding and camera trap surveys as complementary tools for biomonitoring of remote desert water bodies. Environmental DNA, 4, 580–595.

[ece370022-bib-0031] Matteson, S. , Kreitinger, K. , Bartelt, G. , Butcher, G. , Sample, D. , & Will, T. (2009). Partners in Flight Bird Conservation Plan for the boreal hardwood transition (Bird conservation region 12 — U.S. Portion) .

[ece370022-bib-0032] McElreath, R. (2018). Statistical rethinking: A Bayesian course with examples in R and Stan. Chapman Hall/CRC.

[ece370022-bib-0033] McIntyre, T. , Majelantle, T. L. , Slip, D. J. , & Harcourt, R. G. (2020). Quantifying imperfect camera‐trap detection probabilities: Implications for density modelling. Wildlife Research, 47, 177–185.

[ece370022-bib-0034] Mejia, M. P. , Curd, E. , Edalati, K. , Renshaw, M. A. , Dunn, R. , Potter, D. , Fraga, N. , Moore, J. , Saiz, J. , Wayne, R. , & Parker, S. S. (2021). The utility of environmental DNA from sediment and water samples for recovery of observed plant and animal species from four Mojave Desert springs. Environmental DNA, 3, 214–230.

[ece370022-bib-1003] Mena, J. L. , Yagui, H. , Tejeda, V. , Bonifaz, E. , Bellemain, E. , Valentini, A. , Tobler, M. W. , Sanchez‐Vendizu, P. , & Lyet, A. (2021). Environmental DNA metabarcoding as a useful tool for evaluating terrestrial mammal diversity in tropical forests. Ecological Applications, 31, e02335 https://esajournals.onlinelibrary.wiley.com/doi/full/10.1002/eap.2335 33780592 10.1002/eap.2335

[ece370022-bib-0035] Michigan Natural Features Inventory (MNFI) . (2024). Glaucomys sabrinus Northern flying squirrel . Retrieved January 17, 2024, from https://mnfi.anr.msu.edu/species/description/11445/glaucomys‐sabrinus

[ece370022-bib-0036] Pedregosa, F. , Varoquaux, G. , Gramfort, A. , Michel, V. , Thirion, B. , Grisel, O. , Blondel, M. , Prettenhofer, P. , Weiss, R. , Dubourg, V. , Vanderplas, J. , Passos, A. , Cournapeau, D. , Brucher, M. , Perrot, M. , Duchesnay, E. , & Louppe, G. (2011). Scikit‐learn: Machine learning in python. The Journal of Machine Learning Research, 12, 2825–2830.

[ece370022-bib-0037] Perkins, G. C. , Kutt, A. S. , Vanderduys, E. P. , & Perry, J. J. (2016). Evaluating the costs and sampling adequacy of a vertebrate monitoring program. Australian Zoologist, 36, 373–380.

[ece370022-bib-0038] R Core Team . (2022). R: A language and environment for statistical computing. R Foundation for Statistical Computing.

[ece370022-bib-1005] Riaz, T. , Shehzad, W. , Viari, A. , Pompanon, F. , Taberlet, P. , & Coissac, E. (2011). ecoPrimers: Inference of new DNA barcode markers from whole genome sequence analysis. Nucleic Acids Research, 39(21), e145. https://academic.oup.com/nar/article/39/21/e145/1105558 21930509 10.1093/nar/gkr732 PMC3241669

[ece370022-bib-0039] Ryan, E. , Bateman, P. , Fernandes, K. , van der Heyde, M. , & Nevill, P. (2022). eDNA metabarcoding of log hollow sediments and soils highlights the importance of substrate type, frequency of sampling and animal size, for vertebrate species detection. Environmental DNA, 4, 940–953.

[ece370022-bib-1001] Ruppert, K. M. , Kline, R. J. , & Rahman, M. S. (2019). Past, present, and future perspectives of environmental DNA (eDNA) metabarcoding: A systematic review in methods, monitoring, and applications of global eDNA. Global Ecology and Conservation, 17, e00547.

[ece370022-bib-0040] Sales, N. G. , McKenzie, M. B. , Drake, J. , Harper, L. R. , Browett, S. S. , Coscia, I. , Wangensteen, O. S. , Baillie, C. , Bryce, E. , Dawson, D. A. , Ochu, E. , Hänfling, B. , Lawson Handley, L. , Mariani, S. , Lambin, X. , Sutherland, C. , & McDevitt, A. D. (2020). Fishing for mammals: Landscape‐level monitoring of terrestrial and semi‐aquatic communities using eDNA from riverine systems. Journal of Applied Ecology, 57, 707–716.

[ece370022-bib-1004] Sellers, G. S. , Di Muri, C. , Gomez, A. , & Hanfling, B. (2018). Mu‐DNA: A modular universal DNA extraction method adaptablefor a wide range of sample types. Metabarcoding and Metagenomics, 2, 1–11. https://safe.menlosecurity.com/https://mbmg.pensoft.net/articles.php?id=24556

[ece370022-bib-0041] Stein, B. A. , Scott, C. , & Benton, N. (2008). Federal lands and endangered species: The role of military and other federal lands in sustaining biodiversity. Bioscience, 58, 339–347.

[ece370022-bib-0042] Swihart, R. K. , Slade, N. A. , & Bergstrom, B. J. (1988). Relating body size to the rate of home range use in mammals. Ecology, 69, 393–399.

[ece370022-bib-0043] Tabak, M. A. , Norouzzadeh, M. S. , Wolfson, D. W. , Sweeney, S. J. , Vercauteren, K. C. , Snow, N. P. , Halseth, J. M. , Di Salvo, P. A. , Lewis, J. S. , White, M. D. , Teton, B. , Beasley, J. C. , Schlichting, P. E. , Boughton, R. K. , Wight, B. , Newkirk, E. S. , Ivan, J. S. , Odell, E. A. , Brook, R. K. , … Miller, R. S. (2019). Machine learning to classify animal species in camera trap images: Applications in ecology. Methods in Ecology and Evolution, 10, 585–590.

[ece370022-bib-0044] Zentelis, R. , & Lindenmayer, D. (2015). Bombing for biodiversity—Enhancing conservation values of military training areas. Conservation Letters, 8, 299–305.

